# The association between the number of previous episodes and modifiable vulnerability factors in remitted patients with recurrent depression

**DOI:** 10.1371/journal.pone.0206495

**Published:** 2018-11-02

**Authors:** Margo de Jonge, Claudi L. H. Bockting, Patricia van Oppen, Henricus L. Van, Jaap Peen, Martijn J. Kikkert, Jack J. M. Dekker

**Affiliations:** 1 Department of Research, Arkin, Amsterdam, The Netherlands; 2 Department of Clinical Psychiatry, University of Amsterdam, Amsterdam, The Netherlands; 3 Department of Research, GGZ inGeest, Amsterdam, The Netherlands; 4 Department of Clinical Psychology, Vrije Universiteit, Amsterdam, The Netherlands; King's College London, UNITED KINGDOM

## Abstract

**Objective:**

Remitted patients with a history of several previous major depressive episodes have a higher risk of relapse/recurrence than patients with fewer previous episodes, and the probability of another episode increases progressively with each successive episode. This study examines the association between the number of previous episodes and modifiable vulnerability factors in remitted patients with recurrent depression.

**Methods:**

Patients with recurrent depression (DSM-IV-diagnosed) who were in remission (N = 214) were recruited between September 2011 and July 2016. The association was examined between the number of previous episodes and the following factors: i.e. interpersonal functioning, daily stress, sense of mastery, coping and dysfunctional beliefs.

**Results:**

A history of more previous episodes was associated with higher levels of interpersonal problems (P < .001), daily stress (P = .04) and a lower sense of mastery (P = .05). Interpersonal problems were most strongly associated with more previous episodes in a Generalized Linear Regression model. In the domain of interpersonal problems, the subscales that showed the strongest relationship were domineering/controlling, vindictive/self-centred, socially inhibited and self-sacrificing.

**Conclusions:**

Patients with a history of more depressive episodes reported higher levels of interpersonal problems, daily stress and a lower sense of mastery. Future studies should examine these factors in a longitudinal cohort and look at whether the effect of interventions to prevent relapse can be explained by targeting these psychological factors.

**Trial registration:**

Netherlands Trial Register: 2599.

## Introduction

The major contribution of Major Depressive Disorder (MDD) to disability and health care costs is largely due to its highly recurrent nature. Reported recurrence rates for high-risk groups, can be as high as 60–70% over two years [1, 2]. Known illness related factors that contribute to the risk of recurrence are age at onset, depression severity and residual symptoms after remission [3–8]. However, one of the most consistent predictors of recurrence is the number of previous major depressive episodes (MDEs). Remitted patients with more previous episodes have a higher risk of relapse/recurrence than remitted patients with fewer previous episodes, and the probability of another episode increases progressively with each successive episode [6, 9–11]. Explanations that have been given for the high relapse/recurrence risk in MDD are generally based on two hypotheses. First, high relapse/recurrence risk might be accounted for by individual differences in premorbid vulnerability [5, 6]. Second, an explanation for this finding may be found in vulnerability models such as the ‘scarring’ hypothesis, which states that a depressive episode may cause psychological and/or biological scarring that makes the patient more vulnerable to a subsequent episode [5, 6]. The very process of going through an MDE may therefore produce a change in the underlying vulnerability factors that make a new MDE more likely [6, 12]. A study of vulnerability factors (in other words, presumed vulnerability mechanisms where the scars are inflicted) in remitted patients with recurrent depression could therefore identify factors that can be targeted by interventions to prevent relapse.

Several modifiable vulnerability factors have been studied in relation to the recurrence of MDD [5, 6]. Interpersonal problems have been linked to the recurrence of MDD through the stress generation hypothesis [13–16] Several studies show that earlier recurrence can be predicted on the basis of the frequency of daily stress and a more avoidant coping strategy [15, 17, 18]. Furthermore, a cohort study has shown that a low sense of mastery was also a predictor of recurrent depression [19]. Finally, the cognitive model indicates that, activated dysfunctional beliefs are vulnerability factors for relapse/recurrence in depression [20, 21]. However, studies of this area have produced mixed results [22–25]. Even though the factors mentioned here are considered to be vulnerability factors for relapse/recurrence, this has been studied without taking into account the number of previous episodes. For example, we do not know if patients who have had more episodes have more dysfunctional beliefs than patients with less episodes. In other words, the association between these factors and the number of previous MDEs is not clear and the central aim of this study.

This study will explore the association between the number of previous episodes and several factors that previous research has identified as potentially modifiable vulnerability factors in recurrently depressed patients. We will examine whether chronicity in terms of the number of previous MDEs is related to interpersonal problems, daily stress, sense of mastery, coping and dysfunctional cognitions. The number of previous episodes will be the dependent variable and the different factors the independent variables. We expect that a history of more previous MDEs will be associated with higher levels of interpersonal problems, higher levels of daily stress, more avoidant coping, more dysfunctional cognitions and lower levels of sense of mastery.

## Method

This study draws on data from a randomized controlled trial examining the effectiveness of Preventive Cognitive Therapy in the prevention of relapse in recurrent depression [26]. The study was conducted in the Netherlands from September 2011 to July 2016,the main objective being to compare preventive cognitive therapy (PCT) with treatment as usual in the prevention of recurrence in remitted patients with a history of recurrent depression who had received Cognitive Behavioural Therapy (CBT) during the acute phase of the depression. The protocol was approved by the Medical Ethics Committee, Stichting Medische-Ethische Toetsingscommissie Instellingen Geestelijke Gezondheidszorg (METiGG, NL 34721.097.10) and all patients provided informed consent prior to participation. The study was registered with the Netherlands Trial Register http://www.trialregister.nl/trialreg/index.asp (Identifier: 2599). A detailed description of the methods is available elsewhere [26].

### Participants

Participants were recruited through secondary health care facilities and by generating publicity in a range of media. Inclusion criteria were , a) at least two previous MDEs, b) current remission according to DSM-IV criteria for at least 2 months as assessed by the Structured Clinical Interview for DSM-IV Axis I Disorders (SCID-I) [27], c) no, or mild depressive symptoms defined as a current score of <14 on the 17-item Hamilton Depression Rating Scale (HDRS) [28], d) a minimum of eight sessions of CBT during the acute phase of the depression, and e) a command of Dutch that was adequate for participation in the study. Exclusion criteria were a) mania or hypomania or a history of bipolar illness, any psychotic disorder (current and previous), b) current alcohol or drugs abuse, and c) acute predominant anxiety disorder.

### Measures

Following inclusion, patients completed a number of questionnaires. The questionnaires relevant for this study are described below.

#### Previous MDEs, illness-related characteristics

Number of previous MDEs, age of first onset and previous depression severity were determined using the Structured Clinical Interview for DSM-IV Axis I Disorders (SCID-I) [27]. Every past episode was assessed at symptom level, and methods from the Life Chart Interview were used to help patients recall the frequency and duration of lifetime MDEs. Personal landmarks and life anchors were used as memory cues to help assess the specifications of the MDEs.

#### Depressive symptomatology

The 30-item Inventory of Depressive Symptomatology–Self Report (IDS-SR) was used to assess levels of depressive symptomatology. The IDS-SR is a self-report measure which patients use to rate their symptoms on a scale of zero to three over the past 7 days. The IDS-SR rates all DSM-IV core symptom domains including mood, cognitive and psychomotor symptoms, but also commonly associated symptoms including anxiety. The IDS-SR has excellent psychometric properties [29, 30].

#### Interpersonal functioning

The Inventory of Interpersonal Problems (IIP-C) was used to measure interpersonal functioning. This is a self-report measure that consists of 64 items designed to measure interpersonal deficiencies and excesses. Patients are asked to rate two types of items: interpersonal behaviors that are “hard for me to do” (e.g., it is hard for me to be self-confident when I am with other people) and interpersonal behaviors that “I do too much” (for example, I open up to people too much). Items are rated on a five-point response scale ranging from 0 (not at all) to 4 (extremely). The IIP-C has a total score and eight subscales: Domineering/Controlling, Vindictive/Self-centered, Cold/Distant, Socially Inhibited, Non-assertive, Overly Accommodating, Self-Sacrificing and Intrusive/Needy [31]. The Dutch version of the IIP-C has good psychometric properties [32, 33].

#### Daily stress

Daily stress was measured using the 114-item Everyday Problem Checklist (EPCL) [34]. The EPCL is a self-report measure that is designed to measure the frequency and the level of the subjective perception of daily stress over the past two months. It has good psychometric properties [34]. We used the manual to create two scores: the frequency and the intensity of daily stress. The frequency of daily stress was the sum of all items experienced, and it ranged from 0 to 114. The intensity of daily stress reflects the impact of stressors and was calculated by dividing the total intensity of all items by frequency, resulting in a score with a range of 0 (“no impact”) to 3 (“major impact”).

#### Sense of mastery

Mastery was measured using the abbreviated five-item version of the Pearlin Mastery Scale [35]. Mastery is the feeling to which a person perceives himself to be in control of events and ongoing situations around him. Items are rated on a five-point scale ranging from 1 (strongly disagree) to 5 (strongly agree). The score is the sum of the recoded ratings and a higher score therefore indicates a higher sense of mastery.

#### Coping

Coping was measured using two subscales taken from the Dutch ‘Utrechtse Coping Lijst’ (UCL): avoidant coping (8 items) and active approach to problems (7 items). The UCL has good psychometric properties [36, 37]. In the general population, a score for active coping between 15 –and 20 and a score for avoidant coping between 12 and 17 would be considered average [36].

#### Dysfunctional cognitions

Dysfunctional cognitions were measured using the Dysfunctional Attitude Scale (DAS-17). The DAS-17 is the shorter 17-item version of a self-report scale DAS-A designed to measure patterns of negative thinking and beliefs in depressed patients. The DAS-A-17 has good psychometric properties in terms of reliability and convergent construct validity [38]. De Graaf et al. (2009) reported m = 46.3, SD = 14.7 in the general population and m = 68.1, SD = 18.5 in depressed patients.

#### Procedure

The SCID-I was administered at randomization in telephone—or face-to-face interviews conducted by independent trained assessors who attended monthly consensus meetings to enhance inter-rater agreement. The SCID-I interviews were recorded and randomly sampled for rescoring by the other assessors under the supervision of the trainer. The assessors were master students in psychology at the university. The IDS-SR, IIP, EPCL, Mastery, UCL and DAS-17 were administered online, with patient access through a personalized hyperlink. The IDS-SR, IIP, EPCL and Mastery were administered at randomization. The UCL and DAS-17 were administered four weeks later.

#### Data analysis

In this cross-sectional study, the dependent variable (number of previous MDEs) was skewed to the right and it could not be transformed to fit a parametric distribution. To determine if there may be a relationship between the number of previous MDEs and the individual factors, a Spearman’s Rho correlation was done. We then assessed which factor was most strongly associated with the number of previous MDEs. A backward-step Generalized Linear Regression(GLR) Model was used to generate a model for the factors most strongly associated with the number of previous MDEs. This analysis was used because the assumptions for a multiple linear regression model were not met owing to the nonparametric distribution of the dependent variable, the number of previous episodes. All the assumptions for the GLR were met. We calculated the variance inflation factor (VIF) for each subscale of the IIP and they ranged between VIF = 1.01–3.28, indicating no multicollinearity [39]. The first backward-step GLR model included all the measures that were significant in the first analysis as independent variables: Interpersonal Problems, Daily stress frequency, Daily stress intensity and Sense of mastery. The second backward-step GLR model included the same independent variables again and also controlled for residual symptoms, age of onset and time since onset. The third backward-step GLR model was explorative and included all eight subscales of the IIP as independent variables.

Statistical analyses were conducted using SPSS Statistics for Microsoft Windows version 22 [40]. All analyses were two-tailed with a probability level of p < .05.

## Results

A total of 2064 patients from five different treatment centres in the Netherlands were approached between January 2012 and August 2014. A range of media were used to generate awareness of the study. A total of 659 patients were assessed for eligibility: 214 met the inclusion criteria and consented to randomization. Eighty-eight per cent of the included patients were recruited through treatment centres and 12% through the media. An overview of the demographic and clinical characteristics of the participants is presented in Table 1. The average age of the included patients was 43, 32% were male -and the average number of previous episodes was 3.98. This sample profile is comparable with other studies. Scores on the DAS were higher than expected based on previous research (m = 77.9, SD = 17.8) [38].

**Table 1:** Participants’ demographic and clinical characteristics.

Characteristic	N	Descriptive
**Age, mean (SD)**	214	43.41 (11.26)
**Gender, no (%)**	214	
***Male***		68 (31.8)
***Female***		146 (68.2)
**Cohabitating, no (%)**	210	107 (51.0)
**Education, no (%)**	210	
***Lower***		21 (10)
***Intermediate***		67 (31.9)
***Higher***		122 (58.1)
**Previous MDEs, median (IQR)**	214	3 (2)
**Previous MDEs, mean (SD)**	214	3.98 (3.25)
**Previous MDEs, no (%)**	214	
***2 episodes***		75 (35.0)
***3 episodes***		52 (24.3)
***4 episodes***		35 (16.4)
***5 episodes***		18 (8.4)
***6 episodes***		10 (4.7)
***7 episodes***		8 (3.7)
***8+ episodes***		16 (7.5)
**Severity last MDE, no (%)**	213	
***Mild***		23 (10.8)
***Moderate***		105 (49.3)
***Severe***		85 (39.9)
**Age of first onset, mean (SD)**	214	24.57 (11.38)
**Patients on antidepressants, no (%)**	200	57 (28.5)
**Inclusion HDRS, mean (SD)**	214	4.35 (3.68)
**Residual symptoms IDS-SR, mean (SD)**	205	16.98 (9.38)
**Interpersonal Problems, mean (SD)**	202	86.09 (32.47)
**Daily Stress frequency, mean (SD)**	187	35.98 (17.65)
**Daily Stress intensity, mean (SD)**	187	1.08 (0.50)
**Sense of Mastery, mean (SD)**	200	16.70 (3.83)
**Active Coping, mean (SD)**	186	18.30 (3.57)
**Avoidant Coping, mean (SD)**	184	16.80 (3.55)
**Dysfunctional Cognitions, mean (SD)**	188	77.86 (17.79)

*Note*. MDE = Major Depressive Episode, IQR = Interquartile range, Severity last MDE as assessed by Structured Clinical Interview for DSM-IV Axis I Disorders, Mild = 5 symptoms, Moderate = 6–7 symptoms, Severe = 8–9 symptoms, HDRS = 17-item Hamilton Depressive Rating Scale, Residual symptoms IDS-SR = Inventory Depressive Symptomatology- Self Report, Interpersonal Problems = Inventory of Interpersonal Problems total score, Daily Stress = Everyday Problem Check List, Sense of Mastery = Pearlin Mastery Scale, Coping = Utrechtse Coping Lijst, Dysfunctional Cognitions = Dysfunctional Attitude Scale-17.

A relationship was found between the number of previous MDEs and interpersonal problems, frequency and intensity of daily stress and sense of mastery. The results are presented in Table 2. No relationship was found between number of previous MDEs and coping and dysfunctional cognitions.

**Table 2:** Spearman’s rho correlation between previous MDEs and Interpersonal problems, daily stress, mastery, coping and cognitions.

	MDEs		
	N	*r*	p
**Interpersonal Problems**	202	.266	**.000**
**Daily Stress frequency**	187	.150	**.040**
**Daily Stress intensity**	187	.231	**.001**
**Sense of Mastery**	200	.141	**.046**
**Active Coping**	186	-.009	.902
**Avoidant Coping**	184	.058	.438
**Dysfunctional cognitions**	188	-.006	.336

Note: MDE = Major Depressive Episodes, Interpersonal Problems = Inventory of Interpersonal Problems total score, Daily Stress = Everyday Problem Check List, Sense of Mastery = Pearlin Mastery Scale, Coping = Utrechtse Coping Lijst, Dysfunctional cognitions = Dysfunctional Attitude Scale-17.

We then combined the factors for which a significant association had been found in the previous analysis to determine which factors or combination of factors are most strongly associated with more previous MDEs. The analysis showed that having more interpersonal problems was most strongly associated with a higher number of previous episodes. The results are presented in Table 3. We repeated the analysis, controlling for residual symptoms, age of onset and time since onset. Interpersonal problems remained most strongly associated (B = .004, S.E. = .0011, P = .002). Scores for interpersonal problems significantly correlated with the scores for all the other factors that we measured, as well as residual symptoms and age of onset (r ranging from .138 to .425).

**Table 3:** Results of generalized linear regression model analyses for factors significantly associated with the number of previous MDEs.

					95% CI for Exp(B)	
	B	*S*.*E*.	*P*	Exp(B)	Lower	Upper
**Included in model:** **Interpersonal Problems, Daily Stress frequency, Daily Stress intensity, Sense of Mastery**^1^						
Interpersonal Problems	.005	.0011	.000	1.005	1.003	1.007
*Intercept*	.582	.1784	.001	1.789	1.261	2.538
**Included in model:** **Interpersonal Problems subscales**^2^						
Subscale 1: Domineering/Controlling	.032	.0110	.004	1.032	1.010	1.054
Subscale 2: Vindictive/Self-centered	-.031	.0115	.008	.970	.948	.992
Subscale 4: Socially Inhibited	.023	.0080	.005	1.023	1.007	1.039
Subscale 7: Self-sacrificing	.016	.0073	.029	1.016	1.002	1.031
*Intercept*	.548	.1815	.003	1.729	1.212	2.468

Note. MDE = Major Depressive Episodes, Interpersonal Problems = Inventory of Interpersonal Problems, Daily Stress = Everyday Problem Check List, Sense of Mastery = Pearlin Mastery Scale.

^1^Omnibus test; *P* = .000

^**2**^Omnibus test; *P* = .000.

Since we determined interpersonal problems with an instrument that consists of eight subscales, a backward-step generalized linear model was used to see which specific subscales contributed most to the model. This explorative analysis showed that subscales 1, 2, 4 and 7 were most strongly associated with the number of previous episodes. Subscale 1 is ‘Domineering/Controlling’, which means that the subject has more difficulty in relinquishing control over others. Subscale 2 is ‘Vindictive/Self-centred’, which means problems with hostile dominance and the tendency to fight with others. Subscale 4 is ‘Socially Inhibited’, which means the tendency to feel anxious and avoidant in the presence of others. Subscale 7 is ‘Self-Sacrificing’, which means there is a tendency to affiliate excessively. A history of more MDEs was associated with a higher score for ‘Domineering/Controlling’, ‘Socially Inhibited’ and ‘Self-Sacrificing’ but a lower score for ‘Vindictive/Self-centred’.

## Discussion

The central aim of this cross-sectional study was to explore the association between the number of previous episodes and potentially modifiable vulnerability factors in remitted patients with recurrent depression. Our results showed that a history of more MDEs was associated with higher levels of interpersonal problems and daily stress, and a lower sense of mastery. The presence of interpersonal problems was most strongly associated with the number of previous MDEs in a Generalized Linear Regression model. Contrary to our expectations, we found no association between the number of previous MDEs and coping or dysfunctional cognitions.

Our results linking interpersonal problems, daily stress, and sense of mastery to chronicity in terms of the number of previous MDEs are in line with previous research linking these factors to the recurrence of MDD [14, 16, 41–43]. These factors were all individually associated with the number of previous MDEs, indicating that patients with more MDEs have more severe interpersonal problems, more frequent and intense daily stress, and less sense of mastery over their problems. These factors are also associated with each other, as shown by the correlation between them (*r* ranging from .32 to .41).

When we examined the factors in a Generalized Linear Regression model that included interpersonal problems, daily stress and sense of mastery, the presence of interpersonal problems was most strongly associated with chronicity (also after controlling for residual symptoms, age of onset and time since onset). Nevertheless, the association between the IIP and the number of previous episodes was modest as expressed in Exp(B) = 1.005. A previous study has shown that, even though interpersonal functioning improves during treatment for MDE, it does not improve as much as depressive symptoms [44]. Furthermore, a recent study has shown that a deterioration in social-interpersonal functioning preceded and predicted depressive symptoms and even relapse/recurrence [45]. One of the explanations for the effect of interventions to prevent relapse (in other words, MBCT, PCT and Wellbeing CT) could be that they influence interpersonal functioning and therefore reduce daily stress and enhance a sense of mastery. Alternatively, sense of mastery could be improved, resulting in better interpersonal functioning and a reduction in daily stress. Future studies should examine these factors in a longitudinal cohort and determine whether the effect of interventions to prevent relapse can be explained by targeting these psychological factors.

Specific interpersonal problems associated with chronicity were: ‘more difficulty with relinquishing control over others’, ‘feeling more anxious and avoidant in the presence of others’, ‘a tendency to affiliate excessively’ and ‘less hostile dominance’. Given the exploratory nature of these analyses and our cross-sectional study design we are cautious in our interpretation of these results and wish to encourage additional research in this area.

We found no relationship between the number of previous MDEs, active or avoidant coping, and dysfunctional cognitions in this group of remitted patients with recurrent depression. Nevertheless, previous research has provided evidence that higher levels of dysfunctional cognitions and specific types of coping (such as avoidant coping) are risk factors for recurrence [5, 22, 23]. The fact that we found no relationship in this study could be explained by the fact that coping and dysfunctional cognitions are premorbid vulnerability factors that make some patients prone to relapse/recurrence, or that the first MDE may have already caused the change in this specific vulnerability [6]. Another explanation for coping and dysfunctional cognitions not being linked to the number of previous MDEs could be that the effect of coping on recurrence diminishes with an increasing number of previous episodes, as suggested by previous research [17, 46]. Alternatively, coping and dysfunctional cognitions may have been addressed adequately during the CBT in the acute phase of the depression. Another alternative, that applies specifically to dysfunctional beliefs, is that scarring effects on dysfunctional beliefs may only be measurable when the dysfunctional beliefs are activated through negative mood states [22, 47].

This study has several limitations. It included only patients with two or more previous MDEs. It is not known whether the associations found were scarring effects or premorbid characteristics, or the result of the first episodes. Longitudinal studies are needed that study a cohort before the onset of the first episode [6, 12]. Furthermore, we could not examine whether the factors found predicted future relapse and recurrence. In addition, we could not address differences between depressive subtypes. Depression is a heterogeneous disorder and different subtypes may run a different course with regard to relapse/recurrence. Finally, the number of previous MDEs was assessed retrospectively at the start of the study, and recall bias is therefore a possibility. However, research based on retrospective recall has shown that recall bias plays only a minor role [48].

Despite these limitations, this study also has several strengths. We included only recurrently depressed patients who were not depressed upon inclusion. There is a high risk of relapse/recurrence in these patients, but measurements are not affected by current MDEs. We assessed the number of previous episodes with the Structured Clinical Interview for DSM-IV Axis I Disorders, which is considered the ‘gold standard’ for diagnosing MDD. Furthermore, we compared multiple factors that previous studies have shown to be potentially modifiable vulnerability factors. Finally, our results are relevant to clinical practice and they can be used to further improve and evaluate current interventions to prevent relapse.

The present study highlights the association between interpersonal functioning, daily stress and sense of mastery by linking them to chronicity in terms of the number of previous MDEs. However, methodological issues, associated with the cross-sectional nature of this study, preclude the drawing of conclusions about the direction of causality. Current effective interventions for preventing the recurrence of depression could already target these factors directly or indirectly. Studies in the future should therefore examine whether the preventive effects of current interventions can be explained by targeting these factors.

## References

[1] Bockting CL, Spinhoven P, Wouters LF, Koeter MW, Schene AH. Long-term effects of preventive cognitive therapy in recurrent depression: a 5.5-year follow-up study. J Clin Psychiatry. 2009;70(12):1621–8. 10.4088/JCP.08m04784blu 20141705

[2] Vittengl JR, Clark LA, Dunn TW, Jarrett RB. Reducing relapse and recurrence in unipolar depression: a comparative meta-analysis of cognitive-behavioral therapy's effects. J Consult Clin Psychol. 2007;75(3):475–88. 2007-07856-013 [pii]; 10.1037/0022-006X.75.3.475 17563164 PMC2630051

[3] Fava GA, Ruini C, Rafanelli C, Finos L, Conti S, Grandi S. Six-year outcome of cognitive behavior therapy for prevention of recurrent depression. Am J Psychiatry. 2004;161(10):1872–6. 161/10/1872 [pii]; 10.1176/ajp.161.10.1872 15465985

[4] Judd LL, Akiskal HS, Maser JD, Zeller PJ, Endicott J, Coryell W, et al A prospective 12-year study of subsyndromal and syndromal depressive symptoms in unipolar major depressive disorders. Arch Gen Psychiatry. 1998;55(8):694–700. 9707379 10.1001/archpsyc.55.8.694

[5] Bockting CL, Hollon SD, Jarrett RB, Kuyken W, Dobson K. A lifetime approach to major depressive disorder: The contributions of psychological interventions in preventing relapse and recurrence. Clin Psychol Rev. 2015 S0272-7358(15)00022-7 [pii]; 10.1016/j.cpr.2015.02.003 25754289

[6] Burcusa SL, Iacono WG. Risk for recurrence in depression. Clin Psychol Rev. 2007;27(8):959–85. S0272-7358(07)00058-X [pii]; 10.1016/j.cpr.2007.02.005 17448579 PMC2169519

[7] Katon W, Unutzer J, Russo J. Major depression: the importance of clinical characteristics and treatment response to prognosis. Depress Anxiety. 2010;27(1):19–26. 10.1002/da.20613 19798766

[8] Ormel J, Oldehinkel AJ, Nolen WA, Vollebergh W. Psychosocial disability before, during, and after a major depressive episode: a 3-wave population-based study of state, scar, and trait effects. Arch Gen Psychiatry. 2004;61(4):387–92. 10.1001/archpsyc.61.4.387 61/4/387 [pii]. 15066897

[9] Kessing LV, Hansen MG, Andersen PK, Angst J. The predictive effect of episodes on the risk of recurrence in depressive and bipolar disorders—a life-long perspective. Acta Psychiatr Scand. 2004;109(5):339–44. 10.1046/j.1600-0447.2003.00266.x ACP266 [pii]. 15049770

[10] Hardeveld F, Spijker J, De Graaf R, Nolen WA, Beekman AT. Prevalence and predictors of recurrence of major depressive disorder in the adult population. Acta Psychiatr Scand. 2010;122(3):184–91. 10.1111/j.1600-0447.2009.01519.x . 20003092

[11] Hardeveld F, Spijker J, de GR, Nolen WA, Beekman AT. Recurrence of major depressive disorder and its predictors in the general population: results from the Netherlands Mental Health Survey and Incidence Study (NEMESIS). Psychol Med. 2013;43(1):39–48. S0033291712002395 [pii]; 10.1017/S0033291712002395 23111147

[12] Wichers M, Geschwind N, van Os J, Peeters F. Scars in depression: is a conceptual shift necessary to solve the puzzle? Psychol Med. 2010;40(3):359–65. . 20120516 10.1017/s0033291709990420

[13] Hammen C. Generation of stress in the course of unipolar depression. J Abnorm Psychol. 1991;100(4):555–61. 1757669 10.1037//0021-843x.100.4.555

[14] Liu RT. Stress generation: future directions and clinical implications. Clin Psychol Rev. 2013;33(3):406–16. S0272-7358(13)00008-1 [pii]; 10.1016/j.cpr.2013.01.005 23416877

[15] Holahan CJ, Moos RH, Holahan CK, Brennan PL, Schutte KK. Stress generation, avoidance coping, and depressive symptoms: a 10-year model. J Consult Clin Psychol. 2005;73(4):658–66. 2005-11147-009 [pii]; 10.1037/0022-006X.73.4.658 16173853 PMC3035563

[16] Hammen C. Stress generation in depression: reflections on origins, research, and future directions. J Clin Psychol. 2006;62(9):1065–82. 10.1002/jclp.20293 16810666

[17] Bockting CL, Spinhoven P, Koeter MW, Wouters LF, Schene AH, Depression Evaluation Longitudinal Therapy Assessment Study G. Prediction of recurrence in recurrent depression and the influence of consecutive episodes on vulnerability for depression: a 2-year prospective study. J Clin Psychiatry. 2006;67(5):747–55. . 16841624

[18] Bockting CL, Spinhoven P, Koeter MW, Wouters LF, Visser I, Schene AH, et al Differential predictors of response to preventive cognitive therapy in recurrent depression: a 2-year prospective study. Psychother Psychosom. 2006;75(4):229–36. 10.1159/000092893 . 16785772

[19] Colman I, Naicker K, Zeng Y, Ataullahjan A, Senthilselvan A, Patten SB. Predictors of long-term prognosis of depression. CMAJ. 2011;183(17):1969–76. cmaj.110676 [pii]; 10.1503/cmaj.110676 22025655 PMC3225418

[20] Beck AT. Depression New York: Harper and Row; 1967.

[21] Beck AT, Rush AJ, Shaw BF, Emery G. Cognitive therapy of depression New York: Guilford Press; 1979 1979.

[22] van Rijsbergen GD, Bockting CL, Burger H, Spinhoven P, Koeter MW, Ruhe HG, et al Mood reactivity rather than cognitive reactivity is predictive of depressive relapse: a randomized study with 5.5-year follow-up. J Consult Clin Psychol. 2013;81(3):508–17. 10.1037/a0032223 23477478

[23] Jarrett RB, Minhajuddin A, Borman PD, Dunlap L, Segal ZV, Kidner CL, et al Cognitive reactivity, dysfunctional attitudes, and depressive relapse and recurrence in cognitive therapy responders. Behav Res Ther. 2012;50(5):280–6. 10.1016/j.brat.2012.01.008 ; PubMed Central PMCID: PMCPMC3336865. 22445946 PMC3336865

[24] Otto MW, Teachman BA, Cohen LS, Soares CN, Vitonis AF, Harlow BL. Dysfunctional attitudes and episodes of major depression: Predictive validity and temporal stability in never-depressed, depressed, and recovered women. J Abnorm Psychol. 2007;116(3):475–83. 10.1037/0021-843X.116.3.475 . 17696703

[25] Figueroa CA, Ruhe HG, Koeter MW, Spinhoven P, Van der Does W, Bockting CL, et al Cognitive reactivity versus dysfunctional cognitions and the prediction of relapse in recurrent major depressive disorder. J Clin Psychiatry. 2015;76(10):e1306–12. 10.4088/JCP.14m09268 . 26528654

[26] de Jonge M, Bockting CL, Kikkert MJ, Bosmans JE, Dekker JJ. Preventive Cognitive Therapy versus Treatment as Usual in preventing recurrence of depression: protocol of a multi-centered randomized controlled trial. BMC Psychiatry. 2015;15:139 10.1186/s12888-015-0508-8 [pii]. 26129694 PMC4487965

[27] Spitzer RL, Williams JB, Gibbon M, First MB. The Structured Clinical Interview for DSM-III-R (SCID). I: History, rationale, and description. Arch Gen Psychiatry. 1992;49(8):624–9. 1637252 10.1001/archpsyc.1992.01820080032005

[28] Hamilton M. A rating scale for depression. J Neurol Neurosurg Psychiatry. 1960;23:56–62. 14399272 10.1136/jnnp.23.1.56 PMC495331

[29] Rush AJ, Gullion CM, Basco MR, Jarrett RB, Trivedi MH. The Inventory of Depressive Symptomatology (IDS): psychometric properties. Psychol Med. 1996;26(3):477–86. 8733206 10.1017/s0033291700035558

[30] Nolen WA, P.M.A.J. D. Meetinstrumenten bij stemmingsstoornissen. Tijdschrijft voor Psychiatrie. 2004;46(10):681–6.

[31] Alden LE, Wiggins JS, Pincus AL. Construction of circumplex scales for the Inventory of Interpersonal Problems. J Pers Assess. 1990;55(3–4):521–36. 10.1080/00223891.1990.9674088 2280321

[32] Vanheule S, DeSmet M, Rosseel Y. The factorial structure of the Dutch translation of the inventory of interpersonal problems: a test of the long and short versions. Psychol Assess. 2006;18(1):112–7. 2006-03905-014 [pii]; 10.1037/1040-3590.18.1.112 16594820

[33] Horowitz LM, Rosenberg SE, Bartholomew K. Interpersonal problems, attachment styles, and outcome in brief dynamic psychotherapy. J Consult Clin Psychol. 1993;61(4):549–60. 8370851 10.1037//0022-006x.61.4.549

[34] Vingerhoets AJJM, van Tilburg MAL. Everyday Problem Checklist (EPCL). 1994 1994. Report No.

[35] Pearlin LI, Schooler C. The structure of coping. J Health Soc Behav. 1978;19(1):2–21. 649936

[36] Schreuders JPG, van den Willige G, Brosschot JF, Tellegen B, Graus GMH. Herziene handleiding De Utrechtse Coping Lijst: UCL. Omgaan met problemen en gebeurtenissen. 1993 1993. Report No.

[37] Sanderman R, Ormel J. De Utrechtse coping lijst:validiteit en betrouwbaarheid. Gedrag en Gezondheid. 1992;20:32–7.

[38] de Graaf LE, Roelofs J, Huibers MJ. Measuring Dysfunctional Attitudes in the General Population: The Dysfunctional Attitude Scale (form A) Revised. Cognit Ther Res. 2009;33(4):345–55. 10.1007/s10608-009-9229-y 19623267 PMC2712063

[39] Hair JFB, W.C.; Babin B.J.; Anderson R.E.; Tatham R.L. Multivariate Data Analysis NJ: Prentice Hall: Upper Saddle River; 2006.

[40] SPSS Statistics for Windows. 17.0 ed. Chicago: SPSS Inc; 2008.

[41] Daley SE, Hammen C, Davila J, Burge D. Axis II symptomatology, depression, and life stress during the transition from adolescence to adulthood. J Consult Clin Psychol. 1998;66(4):595–603. 9735575 10.1037//0022-006x.66.4.595

[42] Monroe SM, Harkness KL. Life stress, the "kindling" hypothesis, and the recurrence of depression: considerations from a life stress perspective. Psychol Rev. 2005;112(2):417–45. 2005-02750-005 [pii]; 10.1037/0033-295X.112.2.417 15783292

[43] Lewinsohn PM, Allen NB, Seeley JR, Gotlib IH. First onset versus recurrence of depression: differential processes of psychosocial risk. J Abnorm Psychol. 1999;108(3):483–9. 10466272 10.1037//0021-843x.108.3.483

[44] Vittengl JR, Clark LA, Jarrett RB. Improvement in social-interpersonal functioning after cognitive therapy for recurrent depression. Psychol Med. 2004;34(4):643–58. 10.1017/S0033291703001478 ; PubMed Central PMCID: PMCPMC1752239. 15099419 PMC1752239

[45] Vittengl JR, Clark LA, Thase ME, Jarrett RB. Longitudinal social-interpersonal functioning among higher-risk responders to acute-phase cognitive therapy for recurrent major depressive disorder. J Affect Disord. 2016;199:148–56. 10.1016/j.jad.2016.04.017 ; PubMed Central PMCID: PMCPMC4862892. 27104803 PMC4862892

[46] ten Doesschate MC, Bockting CL, Koeter MW, Schene AH. Prediction of recurrence in recurrent depression: a 5.5-year prospective study. J Clin Psychiatry. 2010;71(8):984–91. 10.4088/JCP.08m04858blu 20797379

[47] Elgersma HJ, de Jong PJ, van Rijsbergen GD, Kok GD, Burger H, van der Does W, et al Cognitive reactivity, self-depressed associations, and the recurrence of depression. J Affect Disord. 2015;183:300–9. 10.1016/j.jad.2015.05.018 . 26047308

[48] Wells JE, Horwood LJ. How accurate is recall of key symptoms of depression? A comparison of recall and longitudinal reports. Psychol Med. 2004;34(6):1001–11. . 15554571 10.1017/s0033291703001843

